# Thirty-Day Outcomes after Surgery for Primary Sarcomas of the Extremities: An Analysis of the NSQIP Database

**DOI:** 10.1155/2020/7282846

**Published:** 2020-01-13

**Authors:** Kathryn E. Gallaway, Junho Ahn, Alexandra K. Callan

**Affiliations:** Department of Orthopaedic Surgery, UT Southwestern Medical Center, 1801 Inwood Road, Dallas, TX 75390, USA

## Abstract

**Background:**

Primary bone and soft tissue sarcomas are rare tumors requiring wide surgical resection and reconstruction to achieve local control. Postoperative complications can lead to delays in adjuvant therapy, potentially affecting long-term oncologic outcomes. Understanding postoperative complication risks is essential; however, past studies are limited by small sample sizes.

**Purpose:**

This study uses a large national registry to characterize the incidence of complications and mortality in the first thirty days following surgical management of primary bone and soft tissue sarcomas of the extremities.

**Methods:**

A retrospective review of patients in the National Surgical Quality Improvement Program database was performed. Cases were identified using diagnosis codes for malignant neoplasm of soft tissue or bone and procedure codes for amputation and radical resection. The cohort was subdivided by bone versus soft tissue sarcoma, upper versus lower extremity, and amputation versus limb salvage.

**Results:**

One thousand, one hundred eleven patients were identified. The most frequent complications were surgical site infections, sepsis, and venous thromboembolism. The overall incidence of complications was 14.0%. Unplanned readmission and reoperation occurred after 7.0% and 8.0% of cases, respectively. Thirty-day mortality was 0.3%, with one intraoperative death. Patient factors and complication rates varied by tumor location and surgical modality. Lower extremity cases were associated with higher rates of wound complications and infectious etiologies such as surgical site infections, urinary tract infections, and systemic sepsis. In contrast, patients undergoing amputation were more likely to experience major medical complications including acute renal failure, cardiac arrest, and myocardial infarction.

**Conclusion:**

Approximately 1 in 7 patients will experience a complication in the first thirty days following surgery for primary bone and soft tissue sarcomas of the extremities. The unique risk profiles of lower extremity and amputation cases should be considered during perioperative planning and surveillance.

## 1. Introduction

Primary bone and soft tissue sarcomas are rare tumors accounting for approximately 1% of all new cancer diagnoses in the United States [1]. In adults, the most common sarcomas are undifferentiated pleomorphic sarcoma (UPS) (formerly classified as malignant fibrous histiocytoma (MFH)), liposarcoma, and chondrosarcoma, with osteosarcoma more prevalent in younger adults [2–5]. These tumors are locally aggressive and require accurate diagnosis to provide multidisciplinary care and treatment planning. Surgery is used to establish local control and attempt at cure in cases amenable to resection. In the past, amputation was the preferred surgical modality. With the development of new surgical techniques, advanced imaging, and multimodal therapy, limb-sparing resection can provide equivalent oncologic results while preserving limb function and quality of life [3, 4]. Amputation is reserved for patients with extensive neurovascular involvement precluding limb salvage, prosthetic failure, and palliative treatment for patients with intractable pain or fungating wounds [6–8].

Due to the rare incidence of these tumors, past studies are frequently limited by small sample sizes. National registries and international collaborations have become a cornerstone of orthopedic oncology research [9–11]. These studies focus on epidemiology, tumor recurrence, disease-specific survival, outcomes essential for evaluating treatment protocols, and identifying long-term sequalae. However, oncology databases such as SEER fail to adequately capture patient comorbidities and early postoperative complications [11–13]. Early complications of surgery can delay adjuvant therapy, potentially affecting long-term oncologic outcomes [14]. Therefore, knowledge of risk profiles for specific tumors and procedures is necessary for adequate preoperative planning and postoperative surveillance.

The American College of Surgeons National Surgical Quality Improvement Program (NSQIP) is a national surgical registry prospectively collecting thirty-day perioperative data from more than 600 sites across the United States. The data collection and quality control methodologies for NSQIP have been previously described and shown to be reliable [15, 16]. NSQIP has been used to characterize demographics, comorbidities, and early postoperative complications for primary and metastatic bone tumors of the spine [17, 18] as well as retroperitoneal sarcomas [19]. Additionally, our research team has used NSQIP to evaluate outcomes after surgical management of metastatic tumors of the extremities. To our knowledge, NSQIP has not been used to characterize outcomes after surgery for primary bone and soft tissue sarcomas of the extremities.

The aim of this study is to use a large national registry to characterize the incidence of complications and mortality in the first thirty days following surgical management of primary bone and soft tissue sarcomas of the extremities.

## 2. Methods

### 2.1. Study Design

After receiving approval from the Institutional Review Board, a retrospective review of patient data in NSQIP was performed. Individual Participant Use Files for each year between 2005 and 2017 were combined into a single master file and queried using Apache Zeppelin 0.7.3 (Wakefield, MA). The query was restricted to cases performed between 2011 and 2017 due to changes in readmission and reoperation reporting in NSQIP after 2010.

### 2.2. Cohort Identification

Patients were identified using International Classification of Diseases, Ninth Revision (ICD-9) and Tenth Revision (ICD-10) codes related to malignant neoplasm of bone, connective tissue, soft tissue, and peripheral nerves located in the upper extremity or shoulder (UE) and lower extremity or pelvis (LE). Diagnosis codes used as inclusion criteria are presented in Table 1. This query identified an initial cohort of 2065 patients undergoing surgery for soft tissue or bone sarcomas of the extremities.

**Figure 1:**
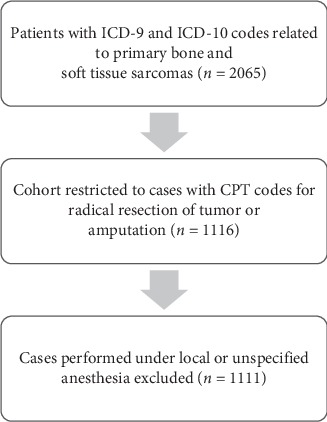
Patient selection process.

Next, the cohort was refined using Current Procedure Terminology (CPT) codes to identify patients undergoing surgery to establish definitive local control. CPT codes used as inclusion criteria include radical resection of bone or soft tissue sarcoma and amputation at any level. Procedure codes used as inclusion criteria are presented in Table 2. This narrowed the cohort to 1116 patients. Finally, cases performed under local or unknown anesthesia were excluded. The final cohort consisted of 1111 cases (Figure 1).

### 2.3. Outcome Measures

Patient demographics, comorbidities, surgical parameters, and thirty-day postoperative outcomes were extracted for analysis. Primary outcome measures were overall complication rate, unplanned readmission, unplanned reoperation, and thirty-day mortality. NSQIP tracks readmission and reoperation as “planned” and “unplanned”. A readmission or reoperation is considered “unplanned” if it is not stipulated in advance as part of the perioperative treatment protocol, such as a staged procedure or adjuvant chemotherapy. Rates of individual complications were analyzed as secondary outcomes. The cohort was partitioned by surgical site (UE versus LE), tumor origin (bone versus soft tissue sarcoma), and surgical modality (amputation versus limb salvage) to determine if there were any differences in patient factors or complication rates.

### 2.4. Statistical Analysis

Descriptive statistics were performed in Apache Zeppelin. Continuous variables were summarized with median and interquartile range values. Categorical variables were reported as frequency counts and percentages. Bivariate analysis was performed in GraphPad Prism 8.0.1 (San Diego, CA) to identify differences between subgroups. Categorical variables were compared using Fisher's exact test and odds ratios (OR). The Haldane-Anscombe correction was applied to OR calculations when the frequency count in a subgroup was zero [20]. Categorical variables with more than two categories were analyzed using Pearson's *χ*^2^ test. Continuous variables were compared using the Mann–Whitney *U* test for nonparametric samples. An *α* value less than 0.05 was considered significant.

## 3. Results

### 3.1. Cohort Characteristics

**Figure 2:**
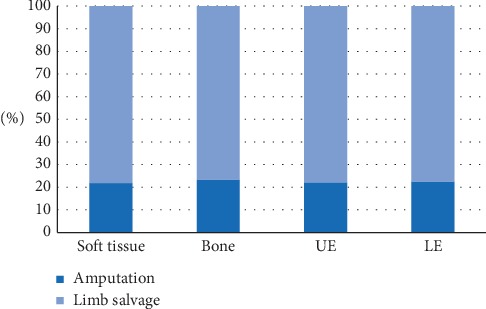
Amputation vs limb salvage procedure with respect to tumor site and origin.

One thousand, one hundred eleven patients were included in our analysis. Soft tissue sarcomas were more common (65.7% soft tissue vs. 34.3% bone), and the majority of tumors were located in the lower extremity (70.3% LE vs. 29.7% UE). Limb salvage procedures were performed in 77.7% of cases with the remaining 22.3% of patients undergoing an amputation. There were no differences in the choice of surgical modality with respect to tumor origin or location (Figure 2).

### 3.2. Demographics and Comorbidities

The median age of the cohort was 58 years (IQR 41–70), and the median BMI was 27.9 (IQR 24.3–32.2). There was a slight male predominance (55.9% male vs. 44.1% female). 71.4% of patients were Caucasian, 8.6% were African American, 4.1% were Asian or Pacific Islander, and 0.7% were American Indian or Alaskan Native. 7.1% of patients were Hispanic. Patients with bone sarcomas were significantly younger (50 vs. 62 years, *p* < 0.0001), had a slightly lower BMI (27.1 vs. 28.1, *p*=0.012), and were more likely to be male (60.9% vs. 53.3%, *p*=0.016). There were no differences in age or BMI with respect to tumor location or surgical procedure. Additionally, there were no differences in race and ethnicity between any subgroups.

NSQIP tracks a number of preoperative comorbidities and risk factors. These preoperative patient factors are summarized in Table 3.

Patients with bone tumors were more likely to be current smokers (OR 1.496, 95% CI: 1.068–2.101), take steroids for a chronic condition (OR 3.315, 95% CI: 1.607–6.669), and have disseminated cancer at the time of surgery (OR 1.720, 95% CI: 1.187–2.515). Soft tissue tumors were associated with higher rates of wound infections present at the time of surgery (OR 2.261, 95% CI: 1.197–4.463) and bleeding disorders (OR 2.278, 95% CI: 1.052–4.815). Lower extremity tumors were more likely to have limited functional status (OR 3.322, 95% CI: 1.333–7.881) or be diagnosed with sepsis or SIRS (OR 2.918, 95% CI: 1.045–7.819). There were no other differences in preoperative comorbidities with respect to tumor origin or location.

Patients undergoing an amputation rather than a limb-sparing procedure had higher ASA classifications, were more likely to be partially or totally dependent, and were more likely to have disseminated cancer at the time of surgery. Additionally, amputation patients were more likely to receive a preoperative transfusion, have an active wound infection, or be diagnosed with preoperative sepsis/SIRS. Significant differences between the amputation and limb salvage groups are presented in Table 4.

### 3.3. Preoperative Labs and Surgical Parameters

Median preoperative lab values were within normal ranges. In patients undergoing an amputation procedure, preoperative lab values were significantly skewed towards abnormal values although median values remained within normal ranges. A summary of preoperative lab values in amputation versus limb salvage patients is presented in Table 5.

Bone sarcomas, lower extremity cases, and limb salvage procedures were associated with significantly longer operation times (*p* < 0.0001 for all subgroup comparisons) and were more likely to be performed on an inpatient basis (OR 3.341, 3.026, and 2.098, respectively). Surgery for bone sarcomas was more likely to be emergent (OR 9.695, 95% CI: 1.341–114.4). Amputation cases were more likely to be performed with monitored anesthesia care (MAC) or regional anesthesia versus general anesthesia (OR 3.018, 95% CI: 1.832–5.004).

### 3.4. Postoperative Complications

The most frequent complications were surgical site infections (SSI), postoperative sepsis or septic shock, venous thromboembolism (VTE), and wound dehiscence. The overall complication rate was 14.0%. Intra- or postoperative bleeding requiring transfusion occurred after 22.3% of cases. The unplanned readmission and reoperation rates were 8.0% and 7.0%, respectively. The thirty-day mortality rate was 0.3% with one intraoperative death. Complication rates for the entire cohort are summarized in Table 6.

Soft tissue sarcomas were associated with a higher rate of superficial SSI (OR 3.432, 95% CI: 1.506–7.603), while bone sarcomas were more likely to experience intra- or postoperative bleeding requiring transfusion (OR 2.517, 95% CI: 1.877–3.342). There were no other differences in complication rates with respect to tumor origin.

Lower extremity and amputation cases were both associated with significantly higher complication rates. Lower extremity cases were more likely to experience infectious and wound complications including superficial and deep SSI, wound dehiscence, urinary tract infections (UTIs), and postoperative sepsis or septic shock. Although all three deaths in this study occurred in lower extremity cases, this finding did not reach statistical significance (*p*=0.559). On the contrary, amputation cases were associated with higher rates of major medical complications including acute renal failure, cardiac arrest requiring CPR, and myocardial infarction. Both lower extremity and amputation cases were associated with higher rates of intra- or postoperative bleeding requiring transfusion. Additionally, they were both associated with an increased likelihood to be discharged to a facility versus home. Differences in complication rates with respect to tumor location and surgical modality are presented in Tables 7 and 8.

## 4. Discussion

Early postoperative complications after surgical resection of bone and soft tissue sarcomas of the extremities are common, with 14.0% of patients experiencing one or more complication in the first thirty days following surgery. Postoperative complication rates reported in recent literature range from 10.6% after resection of low-grade chondrosarcoma [21] to 70.5% after hindquarter amputation [6]. The wide range of reported complication rates reflects the considerable interstudy heterogeneity in the definition of complications, time to assessment, and specific tumor or procedure being evaluated. Few studies report outcomes at the thirty-day postoperative mark despite the potential negative impacts of early postoperative complications on long-term oncologic outcomes.

Studies that do report thirty-day outcomes have similar results to those described in this analysis. Furthermore, these reports show that only 50–60% of complications occur in the first thirty days after surgery [22–24]. A study by Moore et al. reported a 17.6% rate of major wound complications occurring in a median of 21.5 days after soft tissue sarcoma resection [24]. Another study by Puchner et al. demonstrated a 13.6% overall complication rate in the first month, with the complication rate increasing to 29.3% at a median follow-up of 83 months in patients with pelvic sarcomas [23]. Puchner's study also demonstrates that the risk of specific complications evolves during the postoperative period (31% of infectious complications occurred in the first month versus only 15% of mechanical complications). These studies show that postoperative complications continue to be a concern well beyond the early postoperative period. Future prospective studies should aim to record postoperative complication data at regular intervals in order to capture the evolving complication risk profile at each stage of recovery.

Our study found a significantly higher rate of infections and wound complications after treatment of lower extremity sarcomas. This finding supports past studies that found lower extremity tumors were associated with worse outcomes [22, 25]. This difference is largely attributable to anatomic differences such as the proximity of critical neurovascular structures and joint spaces, increasing the difficulty and morbidity of surgical resection [25]. In our study, we found that LE cases were associated with longer operating times (195 vs. 114 minutes, *p* < 0.0001) supporting the idea that LE tumors are larger and require more complex resections. Additionally, longer operative time can potentially increase the likelihood that the patient will be exposed to an infection on the operating table.

Additionally, a study by Moore et al. found that lower extremity tumors were more likely to be larger than 20 cm at diagnosis [24]. The larger the tumor is, the greater the amount of normal tissue becomes compromised, and the larger the radical resection. Larger resections create more dead space that can lead to massive seroma and devitalized tissue. Additionally, in the lower extremity, dependent edema and peripheral vascular disease can significantly impair wound healing. For soft tissue sarcomas in particular, multimodal treatment strategies for large, locally invasive tumors may include neoadjuvant radiotherapy in an attempt to shrink the tumor and maximize the opportunity for en bloc resection [26, 27]. Both bone and soft tissue sarcomas may also employ postoperative radiotherapy for margin-positive resections which are more likely to occur with large tumors or complex anatomic sites [28]. Radiation therapy has been shown to increase the risk of wound healing complications [25, 27, 29], potentially contributing to the difference observed in this study. Unfortunately, NSQIP stopped tracking radiation and chemotherapy in 2014, so we are unable to consider the impact of adjuvant treatment in this analysis.

Another potential risk factor identified in our study is the increased risk of intra- and postoperative bleeding requiring transfusion in LE cases (OR 4.643, 95% CI: 3.048–7.060). Although the association between perioperative blood transfusions and postoperative infections is still under scrutiny, there is some evidence to suggest that immunomodulation after allogenic blood transfusion increases the patient's susceptibility to infection [30, 31]. This is particularly concerning in patients with malignancy who are already in an immunocompromised state. Reducing the need for allogenic blood transfusion with restrictive transfusion thresholds, autologous blood donation, and intraoperative cell salvage is prudent. Additional research is needed to clarify the association between perioperative transfusion and postoperative infection due to the high rate of both events in orthopedic oncology surgery.

Amputation cases were associated with significantly higher rates of major medical complications including acute renal failure, cardiac arrest, and myocardial infarction. This finding most likely reflects differences in the health status of the patients prior to surgery. Amputations were more likely to have limited functional status, higher ASA classification, disseminated cancer, active wound infection, and sepsis/SIRS at the time of surgery, suggesting advanced disease or debilitating complications such as fungating wounds. Furthermore, some amputation patients may have been considered too medically unstable to undergo extensive reconstructive surgery, as indicated by the trend towards abnormal preoperative labs and decreased use of general anesthesia in amputation cases. These findings are generally consistent with contemporary indications for amputation [6–8]. Given these findings, patients requiring an amputation should be closely monitored for systemic complication after surgery.

Interestingly, we found a higher association between patients with metastatic disease undergoing amputation. NSQIP does not distinguish between a patient with metastatic osteosarcoma versus a patient with metastatic colon cancer and a concurrent diagnosis of osteosarcoma. However, the implications for complications and treatment protocols still apply for both groups. In general, we attempt to avoid amputations in patients with incurable metastatic disease because the need for local control no longer supersedes the need to preserve limb function. The increased rate of amputation in patients with metastatic disease highlights the aggressive nature of some sarcomas, obliterating the possibility of limb salvage surgery and progressive metastatic disease. Patients presenting with disseminated disease can be treated medically using bisphosphonates or radiation to control bone pain and limit the progression of disease [32]. They may also undergo conservative surgical procedures such as plate and screw fixation or intramedullary nailing to treat impending or actual pathologic fractures without an aim for complete tumor resection [33]. These patients are not included in our analysis. Therefore, the patients with disseminated disease undergoing an amputation procedure in this study are patients who have failed conservative therapy due to extensive, recalcitrant disease with fungating tumor or intractable pain. This is consistent with the preoperative metastatic presentation observed in this analysis.

## 5. Limitations

This study has several limitations. NSQIP is a surgical database, so it does not capture patients treated nonoperatively. Additionally, NSQIP only tracks patients for the first thirty days after surgery. Complications occurring after this period are not captured, and therefore, long-term complications such as late wound infections, implant failure, delayed bone healing, and local recurrence cannot be evaluated in this study. Some patients may also be lost to follow-up if they present to an outside facility for postoperative care and fail to report back to their surgeon at the NSQIP institution. To address this concern, NSQIP maintains strict due diligence requirements for participating institutions. Clinics must attempt to contact these patients by phone or letter, and participating institutions must maintain a minimum 30-day follow-up rate of 80%. NSQIP is multidisciplinary, so variables are generic by design. There is a lack of granularity with respect to oncology variables such as tumor size, histology, stage, and neoadjuvant or adjuvant therapy. Furthermore, NSQIP does not track tumor predisposition syndromes or molecular susceptibilities of tumors, preventing us from stratifying cases with unique natural histories or treatment considerations. Finally, NSQIP does not contain patient-centered outcome data such as postoperative pain and functional status.

## 6. Conclusion

Approximately 1 in 7 patients will experience postoperative complications in the first thirty days following surgical management of primary bone and soft tissue sarcomas of the extremities. Patients with lower extremity sarcomas have higher rates of infections and wound complications, while patients undergoing amputation are more likely to experience major medical complications. These unique risk profiles should be taken into account when counseling patients about the risks of surgery and planning postoperative surveillance.

## Figures and Tables

**Table 1:** List of diagnosis codes used for patient identification.

ICD-9	ICD-10	Primary sarcomas of bone
170.4	C40.00–0.002	Malignant neoplasm of scapula and long bones of upper limb
170.5	C40.10–0.012	Malignant neoplasm of short bones of upper limb
170.7	C40.20–0.022	Malignant neoplasm of long bones of lower limb
170.8	C40.30–0.032	Malignant neoplasm of short bones of lower limb
170.3	C41.3	Malignant neoplasm of ribs, sternum, and clavicle
170.6	C41.4	Malignant neoplasm of pelvic bones, sacrum, and coccyx

		Primary sarcomas of soft tissue
	C47.10–0.012	Malignant neoplasm of peripheral nerves of upper limb/shoulder
	C47.20–0.022	Malignant neoplasm of peripheral nerves of lower limb/hip
	C47.5	Malignant neoplasm of peripheral nerves of pelvis
171.2	C49.10–0.012	Malignant neoplasm of connective tissue and soft tissue of upper limb/shoulder
171.3	C49.20–0.022	Malignant neoplasm of connective tissue and soft tissue of lower limb/hip
171.6	C49.5	Malignant neoplasm of connective and soft tissue of pelvis

**Table 2:** List of procedure codes used for patient identification.

Radical resection of soft tissue sarcoma							
23077	23078	24077	24079	25077	25078	26117	26118
27049	27059	27329	27364	27615	27616	28046	28047

Radical resection of bone sarcoma							
23200	23210	23220	24150	24152	25170	26250	26260
26262	27075	27076	27077	27078	27365	27640	27641
27645	27646	27647	28171	28173	28175		

Amputation							
23900	23920	24900	24920	24931	25900	25905	25920
25927	26910	26951	26952	27290	27295	27590	27591
27592	27594	27596	27598	27880	27881	27882	27888
27889	28800	28805	28810	28820	28825		

**Table 3:** Preoperative comorbidities and risk factors.

	All patients	Bone sarcoma	Soft tissue sarcoma	*p* value
	*N* = 1111	*N* = 381	*N* = 730	
	*n* (%)	*n* (%)	*n* (%)	
*Functional status*				
Independent	1062 (95.6%)	363 (95.3%)	699 (95.8%)	0.88
Partially dependent	40 (3.6%)	14 (3.7%)	26 (3.6%)	
Totally dependent	3 (0.3%)	1 (0.3%)	2 (0.3%)	
Unknown	6 (0.5%)	3 (0.8%)	3 (0.4%)	

*ASA classification*				
ASA 1	47 (4.2%)	13 (3.4%)	34 (4.7%)	0.738
ASA 2	419 (37.7%)	149 (39.1%)	270 (37.0%)	
ASA 3	596 (53.6%)	202 (53.0%)	394 (54.0%)	
ASA 4	49 (4.4%)	17 (4.5%)	32 (4.4%)	

Current smoker	163 (14.7%)	69 (18.1%)	94 (12.9%)	**0.020**
Disseminated cancer	127 (11.4%)	58 (15.2%)	69 (9.5%)	**0.005**
Diabetes	132 (11.9%)	35 (9.2%)	97 (13.3%)	0.051
Preoperative wound infection	62 (5.6%)	12 (3.1%)	50 (6.8%)	**0.013**
Dyspnea	42 (3.8%)	13 (3.4%)	29 (4.0%)	0.654
Bleeding disorder	42 (3.8%)	8 (2.1%)	34 (4.7%)	**0.045**
Preoperative transfusion	33 (3.0%)	12 (3.1%)	21 (2.9%)	0.853
Steroid use for a chronic condition	127 (2.9%)	20 (5.2%)	12 (1.6%)	**0.001**
COPD	31 (2.8%)	10 (2.6%)	21 (2.9%)	>0.999
Pre-op sepsis/SIRS	31 (2.8%)	13 (3.4%)	18 (2.5%)	0.443
Recent weight loss	26 (2.3%)	9 (2.4%)	17 (2.3%)	>0.999
Congestive heart failure	5 (0.5%)	3 (0.8%)	2 (0.3%)	0.346
Dialysis	5 (0.5%)	2 (0.5%)	3 (0.4%)	>0.999
Ventilator dependent	1 (0.1%)	1 (0.3%)	0 (0.0%)	0.343

**Table 4:** Comorbidities in amputation versus limb salvage patients.

	Amputation	Limb salvage	*p* value
	*N* = 248	*N* = 863	
	*n* (%)	*n* (%)	
*ASA classification*			
ASA 1	3 (1.2%)	44 (5.1%)	**0.044**
ASA 2	91 (36.7%)	328 (38.0%)	
ASA 3	141 (56.9%)	455 (52.7%)	
ASA 4	13 (5.2%)	36 (4.2%)	

			OR (95% CI)

*Functional status*			
Independent	222 (89.5%)	840 (97.3%)	**5.787 (3.160–10.74)**
Partial/total dependence	26 (10.5%)	17 (2.0%)	

Current smoker	47 (19.0%)	116 (13.4%)	**1.506 (1.045–2.164)**
Wound infection	35 (14.1%)	27 (3.1%)	**5.088 (2.988–8.602)**
Preoperative sepsis/SIRS	17 (6.9%)	14 (1.6%)	**4.463 (2.251–9.316)**
Preoperative transfusion	19 (7.7%)	14 (1.6%)	**5.032 (2.479–10.25)**
Disseminated cancer	41 (16.5%)	86 (10.0%)	**1.790 (1.194–2.676)**

**Table 5:** Preoperative labs in amputation versus limb salvage.

	Amputation	Limb salvage	*p* value
	*N* = 248	*N* = 863	
	Median (IQR)	Median (IQR)	
Hematocrit (%)	36.7 (31.7–41.7)	39.0 (35.0–42.4)	**<0.0001**
WBC (10^9/L)	7.6 (5.7–9.5)	6.6 (5.3–8.1)	**<0.0001**
Platelets (10^9/L)	257 (207–346)	243 (198–291)	**0.0027**
INR	1.1 (1.0–1.2)	1.01 (1.0–1.1)	**<0.0001**
PTT (sec)	31.0 (28.1–35.3)	29.9 (27.5–32.3)	**0.002**
Alk. phosphatase (U/L)	88 (71–119)	79 (64–100)	**0.001**
Sodium (mEq/L)	139 (137–141)	140 (138–141)	**0.009**
Albumin (g/dL)	3.8 (3.1–4.2)	4.1 (3.8–4.4)	**<0.0001**
BUN (mg/dL)	14 (10–20)	15 (12–19)	0.328
Creatinine (mg/dL)	0.8 (0.7–1.0)	0.9 (0.7–1.0)	0.232
AST (U/L)	22 (17–31)	22 (18–28)	0.871
Bilirubin (mg/*μ*L)	0.5 (0.3–0.7)	0.5 (0.3–0.6)	0.838

**Table 6:** Postoperative morbidity and mortality.

	All patients	Bone sarcoma	Soft tissue sarcoma	*p* value
	*N* = 1111	*N* = 381	*N* = 730	
	*n* (%)	*n* (%)	*n* (%)	
Superficial SSI	44 (4.0%)	6 (1.6%)	38 (5.2%)	**0.003**
Deep SSI	33 (3.0%)	9 (2.4%)	24 (3.3%)	0.460
Organ space SSI	18 (1.6%)	8 (2.1%)	10 (1.4%)	0.453
Any SSI	91 (8.2%)	23 (6.0%)	68 (9.3%)	0.065
Sepsis/septic shock	31 (2.8%)	13 (3.4%)	18 (2.5%)	0.443
VTE	26 (2.3%)	11 (2.9%)	15 (2.1%)	0.407
Wound dehiscence	20 (1.8%)	8 (2.1%)	12 (1.6%)	0.637
Urinary tract infection	16 (1.4%)	3 (0.8%)	13 (1.8%)	0.288
Pneumonia	12 (1.1%)	6 (1.6%)	6 (0.8%)	0.358
Ventilator > 48 hours	12 (1.1%)	7 (1.8%)	5 (0.7%)	0.122
Unplanned intubation	8 (0.7%)	4 (1.0%)	4 (0.5%)	0.457
Myocardial infarction	6 (0.5%)	1 (0.3%)	5 (0.7%)	0.670
Acute renal failure	4 (0.4%)	1 (0.3%)	3 (0.4%)	>0.999
Renal insufficiency	3 (0.3%)	1 (0.3%)	2 (0.3%)	>0.999
C. diff colitis	3 (0.3%)	0 (0.0%)	3 (0.4%)	0.555
Cardiac arrest	2 (0.2%)	1 (0.3%)	1 (0.1%)	>0.999
Cerebrovascular accident	1 (0.1%)	0 (0.0%)	1 (0.1%)	>0.999
Unlisted complication	7 (0.6%)	2 (0.5%)	5 (0.7%)	>0.999
Overall complication rate	155 (14.0%)	46 (12.1%)	109 (14.9%)	0.203
Bleeding requiring transfusion	248 (22.3%)	127 (33.3%)	121 (16.6%)	**<0.0001**
Unplanned readmission	89 (8.0%)	27 (7.1%)	62 (8.5%)	0.485
Unplanned reoperation	78 (7.0%)	28 (7.3%)	50 (6.8%)	0.805
Death	3 (0.3%)	1 (0.3%)	2 (0.3%)	>0.999

**Table 7:** Postoperative complications in upper versus lower extremity cases.

	Upper extremity	Lower extremity	OR (95% CI)
	*n* (%)	*N* (%)	
Superficial SSI	6 (1.8%)	38 (4.9%)	2.762 (1.210–6.125)
Deep SSI	3 (0.9%)	30 (3.8%)	4.354 (1.464–13.68)
Wound dehiscence	0 (0.0%)	20 (2.6%)	17.79 (2.492–∞)
Urinary tract infection	1 (0.3%)	15 (1.9%)	6.443 (1.101–68.38)
Sepsis/septic shock	3 (0.9%)	28 (3.6%)	4.053 (1.346–12.77)
Overall complication rate	22 (6.7%)	133 (17.0%)	2.873 (1.786–4.620)
Bleeding requiring transfusion	26 (7.9%)	222 (28.4%)	4.643 (3.048–7.060)
Unplanned readmission	18 (5.5%)	71 (9.1%)	1.733 (1.029–2.917)
Discharged to facility	16 (4.8%)	206 (26.4%)	7.031 (4.232–12.09)

**Table 8:** Postoperative complications in amputation versus limb salvage cases.

	Amputation	Limb salvage	OR (95% CI)
	*n* (%)	*n* (%)	
Acute renal failure	3 (1.2%)	1 (0.1%)	10.56 (1.565–137.2)
Cardiac arrest	2 (0.8%)	0 (0.0%)	17.52 (1.613–∞)
Myocardial infarction	4 (1.6%)	2 (0.2%)	7.057 (1.633–37.21)
Overall complication rate	46 (18.5%)	109 (12.6%)	1.575 (1.082–2.286)
Bleeding requiring transfusion	69 (27.8%)	179 (20.7%)	1.473 (1.064–2.019)
Discharge to facility	92 (37.1%)	130 (15.1%)	3.325 (2.415–4.552)

## Data Availability

The perioperative data used to support the findings of this study are included within the article. The surgical database used in this study is available to employees of participating institutions NSQIP Analysis of Primary Sarcoma Surgery through the American College of Surgeons National Surgical Quality Improvement Program. Additional information can be found at https://www.facs.org/quality-programs/acs-nsqip.
